# Experience-dependent development of visual sensitivity in larval zebrafish

**DOI:** 10.1038/s41598-019-54958-6

**Published:** 2019-12-12

**Authors:** Jiaheng Xie, Patricia R. Jusuf, Bang V. Bui, Patrick T. Goodbourn

**Affiliations:** 1 School of Biosciences, The University of Melbourne, Melbourne, Australia; 2 Department of Optometry and Vision Sciences, The University of Melbourne, Melbourne, Australia; 3 Melbourne School of Psychological Sciences, The University of Melbourne, Melbourne, Australia

**Keywords:** Developmental neurogenesis, Sensorimotor processing, Sensory processing, Motion detection, Pattern vision

## Abstract

The zebrafish (*Danio rerio*) is a popular vertebrate model for studying visual development, especially at the larval stage. For many vertebrates, post-natal visual experience is essential to fine-tune visual development, but it is unknown how experience shapes larval zebrafish vision. Zebrafish swim with a moving texture; in the wild, this innate *optomotor response* (OMR) stabilises larvae in moving water, but it can be exploited in the laboratory to assess zebrafish visual function. Here, we compared spatial-frequency tuning inferred from OMR between visually naïve and experienced larvae from 5 to 7 days post-fertilisation. We also examined development of synaptic connections between neurons by quantifying post-synaptic density 95 (PSD-95) in larval retinae. PSD-95 is closely associated with *N*-methyl-D-aspartate (NMDA) receptors, the neurotransmitter-receptor proteins underlying experience-dependent visual development. We found that rather than following an experience-independent genetic programme, developmental changes in visual spatial-frequency tuning at the larval stage required visual experience. Exposure to motion evoking OMR yielded no greater improvement than exposure to static form, suggesting that increased sensitivity as indexed by OMR was driven not by motor practice but by visual experience itself. PSD-95 density varied with visual sensitivity, suggesting that experience may have up-regulated clustering of PSD-95 for synaptic maturation in visual development.

## Introduction

Experience plays an important role in the early visual development of vertebrates^1–6^. For example, visual stimulation is required for the monostratification of retinal ganglion cell (RGC) dendrites in the inner plexiform layer (IPL), which contributes to the maturation of *ON* and *OFF* visual pathways in the mouse retina^1^. In ferrets, the emergence of cortical orientation selectivity is driven by early experiences with moving visual stimuli, enhancing sensitivity to their spatiotemporal features^2^. However, not all post-natal visual development depends on experience. While visual evoked binocular responses emerge at a similar post-natal age in both pre-term and full-term human infants (suggesting experience-dependence)^6^, monocular responses instead track time from conception, suggesting development independent of experience. The role of visual experience in larval zebrafish visual development has been controversial. Some previous studies have suggested that visual experience may not be necessary for early structural and functional development of some visual regions (e.g., tectum and retina) in larval zebrafish^7,8^. However, a recent study indicates that visual experience contributes to the reorganisation of functional circuits and shapes spontaneous neural activity in the developing larval tectum, and is crucial for the development of prey-capture behaviour^9^.

The molecular mechanisms underlying experience-dependent visual development are also coming to light. Up-regulated expression of brain-derived neurotrophic factor (BDNF) has been observed in *Xenopus* tadpole tectal neurons after exposure to visual stimulation, accompanied by increased visual acuity^10^. Activity-dependent expression of BDNF requires appropriate activation of *N*-methyl-D-aspartate (NMDA) receptors, α-amino-3-hydroxy-5-methyl-4-isoxazolepropionic acid (AMPA) receptors and γ-Aminobutyric acid A (GABA_A_) receptors in the developing visual cortex^10–13^. Excitatory NMDA and AMPA receptors interact with the scaffold protein post-synaptic density 95 (PSD-95) to form surface complexes that anchor the receptors on post-synaptic membranes for signalling^14,15^. During development, PSD-95 contributes to the maturation of excitatory synapses and stabilises synaptic contacts^16,17^. Expression of PSD-95 is up-regulated in the post-synaptic membrane of neurons in human visual cortex during childhood, pointing to an important role in early visual development^18^.

The zebrafish (*Danio rerio*) has become a popular vertebrate model in visual neuroscience. Its high conservation of genetics and retinal architecture with other vertebrate species make the zebrafish ideal for modelling the human visual system^19^. A prolific breeder, the zebrafish produces embryos that develop outside the mother and are transparent until 7 days post-fertilisation (dpf), allowing real-time observation of neurogenesis inside the embryonic body^20–22^. Thus, the early morphological development of the larval zebrafish visual system, including the retina, has been well documented^19,21,22^. In comparison, relatively few studies to our knowledge have characterised the functional, behavioural aspect of visual development in larval zebrafish^23–26^. The zebrafish visual system is behaviourally competent as early as 68 hours post-fertilisation (hpf)^25^, and the *optomotor response* (OMR) can be elicited from 4 dpf^27^. In the wild, larval zebrafish will follow a moving texture to stabilise themselves in the river bed; this OMR is often exploited in the lab as a behavioural screen for visual deficits^24,27^ or, less commonly, to characterise the spatiotemporal or chromatic response properties of the visual system^28,29^. Although studies of zebrafish neuroanatomy largely focus on the early developmental stages—especially those ages at which external food is not required (<7dpf)—the corresponding development of behavioural sensitivity as indexed by OMR has not yet been profiled. Further, the role of visual experience in larval zebrafish visual development and its underlying molecular mechanisms remain to be fully described.

Here, we report that visual experience accelerates development of visual sensitivity to contrast inferred from OMR in larval zebrafish from 5 to 7 dpf. We demonstrate that exposure to static visual form, which does not elicit the OMR, is sufficient to induce experience-dependent improvements in sensitivity; this indicates that the increased OMR reflects visual, rather than motor, development. In addition, we find that PSD-95 density in the retinal IPL increases with visual experience during this developmental period. These results reveal highly experience-dependent development of visual sensitivity at the behavioural level and point to a potential biomolecular mechanism.

## Methods

### Animal husbandry

Zebrafish (wild type St Kilda or AB strains) were bred and housed in the facility at the Walter and Eliza Hall Institute of Medical Research in accordance with local animal guidelines. Male and female zebrafish embryos were raised in egg water (60 mg/L sea salt) at 28.5 °C and reared in complete darkness when not exposed to visual stimulation within the experimental protocol. All procedures were performed according to the provisions of the Australian National Health and Medical Research Council code of practice for the care and use of animals, and were approved by the Faculty of Science Animal Ethics Committee at the University of Melbourne (No. 1614017.3).

### Optomotor apparatus

**Figure 1:**
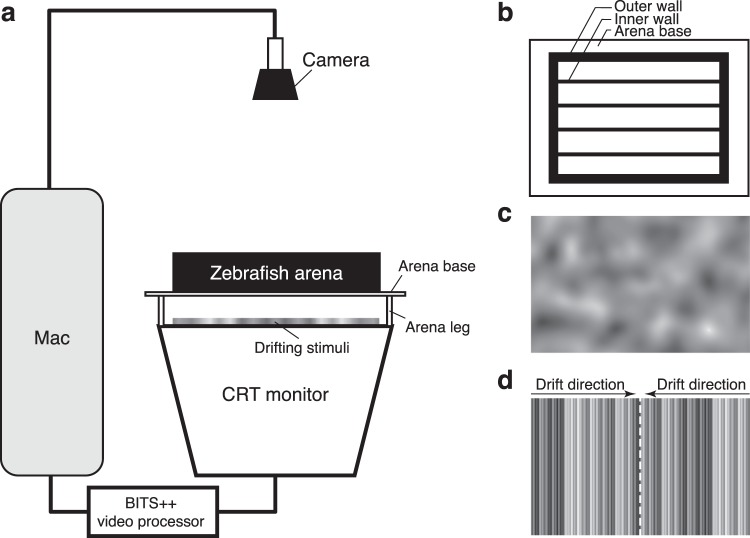
Schematic of the optomotor assay. (**a**) Stimuli were passed through a BITS++ video processor and displayed on a flat-screen cathode ray tube (CRT) monitor facing upwards. The five-lane zebrafish arena with a transparent base was positioned above the monitor screen, and a camera fixed above the arena obtained digital images of larvae positions before and after each trial. (**b**) Overhead view of the zebrafish arena, with inner walls dividing the arena into multiple lanes. (**c**) Stimuli were narrow-band filtered Gaussian-noise textures drifting parallel to the long axis of the arena lanes. (**d**) Between trials, larvae were corralled to the centre of the lane by high-contrast compound gratings drifting from each end, converging at the midline (dashed white line).

The OMR apparatus was the same as described in our previous work^30^, adapted from one used by Orger and colleagues (Fig. 1a,b)^27^. A Power Mac G5 computer (Apple Computer, Inc., Cupertino, CA) ran MATLAB 2016b (MathWorks, Natick, MA) with Psychtoolbox extensions^31^. Stimuli were processed on an ATI Radeon HD 5770 graphics card, with the output sent to a BITS++ video processor (Cambridge Research Systems, Rochester, UK) for increased contrast resolution, and displayed on a M992 flat-screen cathode ray tube (CRT) monitor (Dell Computer Corporation, Round Rock, Texas, USA) with its screen facing upwards. During the experiment, zebrafish were contained in a five-lane arena (300 mm × 38 mm per lane; Fig. 1b) with a transparent base positioned 56.5 mm above the screen. A C922 Pro Stream webcam (Logitech Company, Lausanne, Switzerland; 1080p at 30 Hz) was fixed 366.5 mm above the base of the arena, controlled by the MATLAB program to take digital images (1080 × 1920 pixels) before and after each trial for calculation of swimming distance.

### Optomotor response measurements

After collection, zebrafish embryos were randomly assigned to one of five exposure regimes (Table 1). There were 4–7 groups per regime, with 50–60 larvae in each group; thus, in total, there were over 200 larvae tested for each regime. Some measurements were derived from the same larvae at different timepoints; for example, measurements from larvae in Regime 2 at 6 dpf are referred to as ‘6-dpf naïve’ in the first analysis (*experience-dependent development of visual sensitivity*), while measurements from the same larvae at 7 dpf are referred to as ‘7-dpf 1-session moving’ in the second analysis (*exposure to visual form versus motion*).

**Table 1:** Protocol for optomotor response measurements.

Age	5 dpf	6 dpf	7 dpf
**Regime 1 (7 groups)**			
Condition	Moving stimuli	Moving stimuli	Moving stimuli
Group name (analysis 1)*	5-dpf naïve	6-dpf experienced	7-dpf experienced
Group name (analysis 2)**	—	—	7-dpf 2-session moving
**Regime 2 (5 groups)**			
Condition	No exposure	Moving stimuli	Moving stimuli
Group name (analysis 1)	—	6-dpf naïve	—
Group name (analysis 2)	—	—	7-dpf 1-session moving
**Regime 3 (5 groups)**			
Condition	No exposure	No exposure	Moving stimuli
Group name (analysis 1)	—	—	7-dpf naïve
Group name (analysis 2)	—	—	
**Regime 4 (5 groups)**			
Condition	Static stimuli	Static stimuli	Moving stimuli
Group name (analysis 1)	—	—	—
Group name (analysis 2)	—	—	7-dpf 2-session static
**Regime 5 (4 groups)**			
Condition	No exposure	Static stimuli	Moving stimuli
Group name (analysis 1)	—	—	—
Group name (analysis 2)	—	—	7-dpf 1-session static

*Analysis 1: Experience-dependent development of visual sensitivity. **Analysis 2: Exposure to visual form versus motion.

On the day of an optomotor assay or static exposure, each group was transferred to one of the arena lanes. The transfer period took less than 10 minutes, after which all larvae were allowed to adapt to the arena for 10 minutes. Stimuli were random Gaussian-noise textures presented at 100% contrast (Fig. 1c). These were filtered to be spatial-frequency bandpass and isotropic using log-Gabor filters^32^. Contrast was flattened to remove large-scale fluctuations across the texture^33,34^. Centre frequencies were 0.005, 0.01, 0.02, 0.04, 0.08, 0.16 or 0.32 cycles per degree (c/°) with a standard deviation of 0.5 octaves. Velocities for moving stimuli were 25, 50 or 100 degrees per second (°/s), equivalent to 24.7, 49.3 and 98.6 mm/s in our setup, parallel to the long axis of the arena.

Prior to each trial, a corralling stimulus was shown for 30 s to guide larvae to the centre of the lane (Fig. 1d). This stimulus comprised several sinusoidal luminance gratings with spatial frequencies intermediate to those presented during the trials, superposed with randomised phases. One compound grating drifted from each end of the lane, converging on a central line (Fig. 1d). The corralling stimulus drifted at 25 °/s for sessions with moving stimuli and was static for sessions with static stimuli. This was followed by a 30-s presentation of a test stimulus. Images were taken by the camera before and after each presentation to capture the position of each larva. A blank grey screen was presented after the offset of the test stimulus as the texture for the next trial was computed. Each of the groups underwent no more than one session per day. In sessions with moving stimuli, each combination of spatial frequency and velocity was presented 6 times (3 times in each direction). In sessions with static stimuli, larvae were exposed to each spatial frequency 18 times. Trials were presented in random order. Each experimental session comprised a total of 126 trials and took about 3 h. After each session, larvae were immediately transferred back to dark incubators, or humanely killed in 0.1% tricaine at 7 dpf. All experiments were conducted between 9:30 a.m. and 6:00 p.m.

### Histological processing and immunohistochemistry

Following humane killing in 0.1% tricaine, zebrafish embryos were fixed in 4% paraformaldehyde (PFA) in phosphate-buffered saline (PBS), cryoprotected in 30% sucrose in PBS solution, embedded in OCT (Tissue-Tek) and cryostat sectioned at 12 µm thickness. Antibody staining was carried out at room temperature using standard protocols. Antigen retrieval was performed by incubating slides in boiled 10 mM sodium citrate (pH 6) until the solution was cooled down to room temperature. Slides were then blocked in 5% foetal bovine serum (FBS) for 30 min and incubated overnight in rabbit anti-PSD95 antibody (1:100, Abcam, cat. number ab18258) diluted in the same block solution. On the second day, slides were rinsed using PBS and then incubated for 2 h in anti-Rabbit Alexa Fluor 488 (1:500, Thermo Fisher Scientific, cat. number A11001) diluted in 5% FBS. Nuclei were then stained with 4′,6-diamidino-2-phenylindole (DAPI; 1:10000, Sigma-Aldrich, cat. number D9542-10MG) in PBS solution. Sections were mounted in Mowiol (Sigma-Aldrich, cat. number 81381-250 G).

### Retinal image acquisition

Stained retinal sections were imaged using an A1R confocal microscope (Nikon Corporation, Tokyo, Japan; 60× oil objective, NA = 1.4; excitation wavelengths 405, 488, 561 and 640 nm). The *deconvolution* function was used to enhance PSD-95 puncta against the staining background. Image brightness and contrast were adjusted using FIJI^35^ or Photoshop (Adobe, San Jose, CA, USA) software.

### Optomotor analysis

Larval positions were extracted from the before and after images for each trial using a MATLAB algorithm. Image contrast was flattened, and a Laplacian-of-Gaussian was applied to highlight edges^33^. Thresholding was then applied (and manually adjusted if necessary) to segment larvae from the background, and the spatial centroid of the whole group was calculated using *bwconncomp* and *regionprops* commands from the MATLAB Image Processing Toolbox. On each trial, the change in position of the centroid in the direction of the texture motion was taken as the optomotor index. Within each stimulus speed, normalised optomotor indices were calculated by normalising data to the mean index at 0.02 c/° for 5 dpf larvae (for analysis of OMR development from 5 to 7 dpf) or to the index at 0.02 c/° for 7 dpf 2-session moving larvae (for the analysis of 7 dpf larval OMR as a function of visual experience). Following normalisation of measurements within each velocity, the optomotor index is equivalent to normalised gain (calculated as the ratio of swimming speed to stimulus speed) reported in some larval OMR studies. These linear transformations had no effect on the model fitting or statistical analysis but converted OMR indices into more readily interpretable units. The spatial-frequency tuning function was fitted as a log-Gaussian using a least-squares criterion. For each fitted model, the estimated parameters were amplitude (i.e., the height of the peak), peak frequency (i.e., the spatial frequency at which amplitude peaked) and bandwidth (i.e., the standard deviation; Supplementary Fig. S1). To determine whether parameter estimates differed between groups, we used an *F* test to compare a full model, in which each group’s estimates could vary independently, with a restricted model, in which one parameter was constrained to be the same across groups^36^. We applied Bonferroni correction to the set of pairwise comparisons across groups within a parameter (α = 0.05; α_corrected_ = 0.005). Reported *P* values are adjusted accordingly. There were between 24 and 42 trials per data point in each exposure condition (see Supplementary Tables S1 and S2).

### Histological analysis

One randomly selected region of interest of the IPL (10 × 20 µm) was cropped from images of each retinal section using Photoshop software. The density of PSD-95 puncta in the IPL was then quantified using the *Analyse particles* function in FIJI^35^. Bayesian one-way ANOVA was performed using JASP^37^ to compare PSD-95 density between groups. There were between 10 and 12 retinae per group (see Supplementary Table S3).

## Results

### Experience-dependent development of visual sensitivity

**Figure 2:**
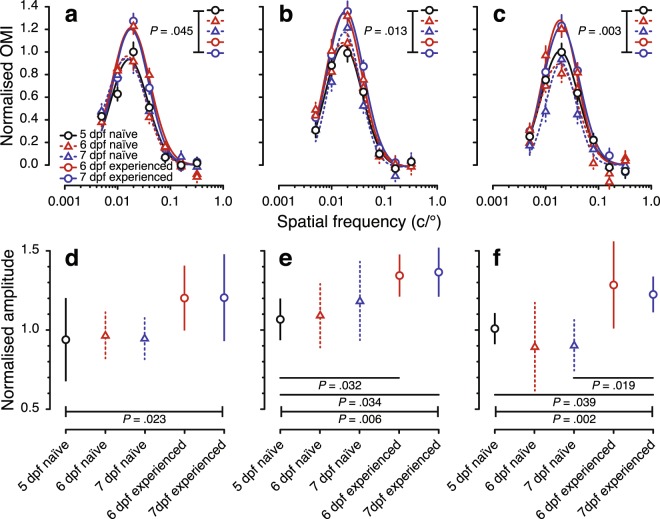
Spatial-frequency tuning from the optomotor response of visually naïve and experienced larval zebrafish from 5 to 7 dpf. The top row shows spatial-frequency tuning functions (mean ± SEM) at stimulus speeds of (**a**) 25 °/s (**b**), 50 °/s, and (**c**) 100 °/s. Black solid, red dashed and blue dashed lines show tuning functions for visually naïve 5, 6 and 7 dpf larvae, respectively. Red and blue solid lines show spatial-frequency tuning functions for visually experienced 6 and 7 dpf larvae, respectively. Functions are a three-parameter log-Gaussian, fit by minimising the least-squares error. The bottom row shows normalised amplitude at stimulus speeds of (**d**) 25 °/s (**e**), 50 °/s, and (**f**) 100 °/s. Error bars show the 95% confidence intervals. *P* < 0.05 after Bonferroni correction was considered to be statistically significant.

To investigate whether visual experience affects early development of visual behaviour in larval zebrafish, we measured the spatial-frequency tuning function using OMR in visually naïve (i.e., dark-reared before measurements) and experienced (i.e., exposed to texture before measurements) larvae from 5 to 7 dpf. All spatial-frequency tuning functions were well fit by the spatially bandpass log-Gaussian model (Fig. 2a–c). An omnibus test indicated that tuning functions differed between groups for each speed (*P* = 0.045, *P* = 0.013 and *P* = 0.003 for 25, 50 and 100 °/s, respectively; Supplementary Table S1).

Examining individual parameters, visual experience had no discernible effect on peak frequencies or bandwidth at any stimulus speed (Supplementary Fig. S2). However, as can be seen in the fitted tuning curves (Fig. 2a–c), amplitude differed between groups at each stimulus speed. At 25 °/s (Fig. 2d), there was overall difference across groups (*P* = 0.023), although no individual pairwise comparison survived Bonferroni correction (Supplementary Table S1). At 50 °/s (Fig. 2e), there was an overall effect of group on amplitude (*P* = 0.006); pairwise comparisons revealed that the amplitude of the tuning function for 5 dpf larvae was lower than for 6 dpf (*P* = 0.032) and 7 dpf (*P* = 0.034) experienced larvae, but indistinguishable from 6 and 7 dpf naïve larvae. Similarly, at 100 °/s (Fig. 2f), there was an overall effect of group on amplitude (*P* = 0.002); amplitude was higher for 7 dpf experienced larvae than for 5 dpf larvae and 7 dpf naïve larvae (*P* = 0.039 and 0.019, respectively; Fig. 2f). Overall, visual experience was necessary for improvements in sensitivity from 5 to 7 dpf.

### Exposure to visual form versus motion

Some proportion of the increased OMR in visually experienced larvae might be attributable to enhanced motor function from repeated stimulation of the response on previous days. In order to assess this possibility, we measured the spatial-frequency tuning function using OMR in 7 dpf larvae with different levels of previous experience (0, 1 or 2 sessions) of either static or moving stimuli. The static stimulus provided exposure to form vision without eliciting the OMR, while moving stimulus evoked a motor response.

**Figure 3:**
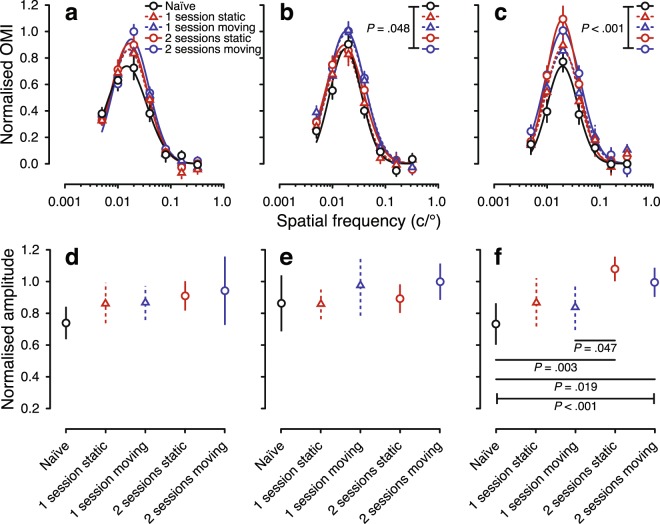
Spatial-frequency tuning from the optomotor response of visually naïve and experienced larval zebrafish at 7 dpf. The top row shows spatial-frequency tuning functions (mean ± SEM) at stimulus speeds of (**a**) 25 °/s (**b**), 50 °/s, and (**c**) 100 °/s. Black solid, red dashed, blue dashed, red solid and blue solid lines show functions for naïve, 1-session static, 1-session moving, 2-session static and 2-session moving groups, respectively. Tuning functions are a three-parameter log-Gaussian, fit by minimising the least-squares error. The bottom row shows normalised amplitude at stimulus speeds of (**d**) 25 °/s (**e**), 50 °/s, and (**f**) 100 °/s. Error bars show the 95% confidence intervals. *P* < 0.05 after Bonferroni correction was considered to be statistically significant.

Inspection of the spatial-frequency tuning functions (Fig. 3a–c) suggests that sensitivity in all experienced 7 dpf groups was higher than that of 7 dpf naïve larvae, with little difference between static and moving exposure groups. This observation was generally supported by formal comparison of the models (Supplementary Table S2). While models were not statistically different from each other at 25 °/s (Fig. 3a), they differed across groups at 50 °/s (*P* = 0.048; Fig. 3b) and 100 °/s (*P* < 0.001; Fig. 3c). Examining individual parameters, visual experience had no discernible effect on peak frequencies or bandwidth at any stimulus speed (Supplementary Fig. S3). At slower speeds, amplitude was numerically (but not significantly) higher in experienced groups than in the naïve group (Fig. 3d,e); but there was a statistical difference in amplitude between groups at 100 °/s (*P* < 0.001; Fig. 3f). The amplitude of the tuning function for the 2-session moving group was significantly higher than that of the naïve group (*P* = 0.019), and significantly higher for the 2-session static group than for both the 1-session moving group (*P* = 0.047) and the naïve group (*P* = 0.003). There was no significant difference in amplitude between groups with moving versus static stimulus experience. This suggests that the primary driver of increased OMR in experienced larvae was not motor development, but rather the visual experience itself.

### Synaptic density in the inner plexiform layer

Our behavioural data demonstrate experience-dependent development of the larval zebrafish OMR from 5 to 7 dpf. Thus, we investigated whether differential development over this period is reflected in different levels of synaptic maturation. PSD-95 is known to play an important role in neuronal signalling by anchoring excitatory NMDA neurotransmitter receptors on the post-synaptic neural membrane during early synaptic development^14,15,17^. The retina is responsible for sensation and initial processing of visual inputs. In zebrafish, PSD-95 is expressed throughout the retina, but the inner plexiform layer (IPL) is particularly suited to quantification of PSD-95 density owing to an absence of cell bodies—bipolar (BC) and amacrine cells (AC) terminate in the IPL to contact ganglion cell (GC) dendrites^38^, making PSD-95 puncta much easier to observe. We thus measured PSD-95 density in the retinal IPL as a marker of synaptic maturation.

**Figure 4:**
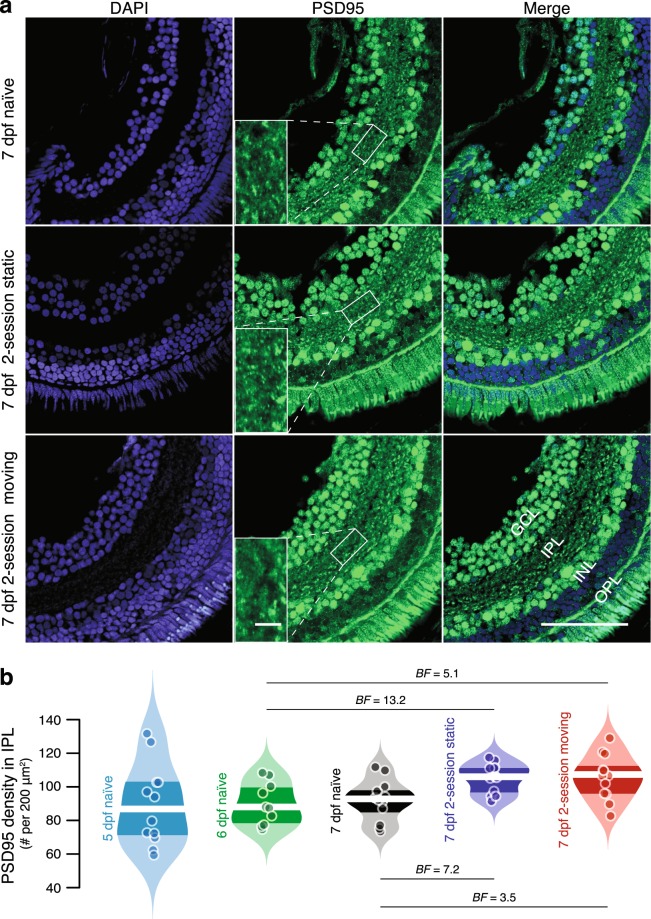
PSD-95 in the retinal IPL of visually naïve and experienced larvae from 5 to 7 dpf. (**a**) Example confocal images of 7 dpf retinae stained with DAPI (blue) and PSD-95 (green) in naïve (top row), 2-session static experienced (middle row) and 2-session moving experienced (bottom row) larvae. The larger scale bar (bottom right) represents 50 µm. Example cropped images for puncta analysis are also shown in the middle column, with the smaller scale bar representing 5 µm. Sections show the ganglion cell layer (GCL), inner nuclear layer (INL), inner plexiform layer (IPL), and outer plexiform layer (OPL). (**b**) Violin plots of PSD-95 density in 5 to 7 dpf visually naïve and experienced larvae. Points are individual retinae, white lines represent medians and dark bands indicate the inter-quartile range. Bayes Factors (*BF*) >3, considered as evidence in favour of a difference between groups, are shown.

PSD-95 puncta were spread throughout the IPL of both naïve and experienced larvae from 5 to 7 dpf (Fig. 4a). However, quantification revealed that both 2-session static and 2-session moving groups at 7 dpf had higher PSD-95 densities than 6- and 7-dpf naïve groups (Fig. 4b, Supplementary Table S3). This raises the possibility that experience-dependent development of visual sensitivity is reflected at a molecular level in improved communication between synapses. In contrast, we found no evidence of a difference in PSD-95 density between 2-session static and 2-session moving 7-dpf larvae. While there was no evidence of a difference between the 5-dpf naïve group and other groups, this is likely a result of highly variable PSD-95 density at 5 dpf.

## Discussion

This study demonstrates experience-dependent visual development in larval zebrafish from 5 to 7 dpf using the optomotor response (OMR). Larval zebrafish have several innate responses, including OMR, that can be used to study visual function^27,39^. Due to the advantages of the zebrafish model and continuing refinement of setups for behavioural measurement, larval zebrafish behavioural assays have been increasingly popular in drug and genetic screening^27,40,41^. In spite of this, much remains to be learned regarding the basic development of visual behaviour in larval zebrafish. We recently showed developmental changes in the larval zebrafish electroretinogram (ERG)^42^, with a considerable acceleration of neural responses generated by retinal photoreceptors and bipolar cells from 4 to 7 dpf. However, development of the larval OMR, its interaction with visual experience, and its subcellular correlates have not been investigated previously.

Zebrafish perform visual behaviours with an immature visual system, which requires visual experience for further maturation. Our data show significant increases in the amplitude of the spatial-frequency tuning function measured by OMR between 5 and 7 dpf in visually experienced larvae, with little discernible change in amplitude in naïve larvae (Fig. 2). This indicates that exposure to visual stimulation at the larval stage may be necessary for maturation of visual behaviour. In many mammals, such as humans, mice and cats, there is a critical period after eye opening during which the neural circuits of the immature visual system are refined^3,12^. During this period, the development of response selectivity in visual neurons depends on experience^1,2^; without appropriate experience (e.g., if the animal is dark-reared), the visual cortex remains immature, manifesting in lower visual sensitivity^3,43^. However, this is not so clear in the case of zebrafish, for which visual development is often considered to be hardwired. For example, the refinement of neural circuits in the optic tectum is almost complete at 78 hpf in dark-reared larvae, prior to the arborisation of retinal axons in the tectum^7,8^. Whether visual development in larval zebrafish is activity-dependent remains controversial^7,44^; however, physiological studies suggest that zebrafish larvae reared under dark or restricted spectral conditions manifest altered retinal functions, consistent with activity-dependent development^45,46^. A critical period at 5–6 dpf for larval zebrafish was also indicated by a recent study, which found that visual experience at this age was critical for the development of larval optic tectum and prey-capture behaviour^9^. Our study adds to this growing body of evidence for experience-dependent visual development in larval zebrafish.

The importance of exposure to light and form for early visual development has been indicated previously in several vertebrate species^1,24,47–49^. Our study extends these findings to show that exposure to visual form is the driving factor in the development of visual sensitivity at the larval stage. By assessing OMR in experienced 7-dpf larvae, we found that improvements in visual sensitivity were similar for larvae exposed to static and moving stimuli. This was clearest at the highest stimulus speed tested (100 °/s), at which improvements were more statistically robust than at the lowest speed (25 °/s). Unequal development of motion sensitivity across different stimulus speeds has been shown in human studies^50^, and on this basis we suspect that in zebrafish larvae, experience may improve visual sensitivity at higher stimulus speeds at a faster developmental rate.

Notably, experience of moving stimuli was not necessary to produce improvements in sensitivity. Exposure to moving stimuli at early ages contributes to development of circuits for direction selectivity, shown in developing ferret visual cortex and *Xenopus* tectal neurons^2,51^. Of course, in our experiment, larvae with only static exposure would have experienced some visual motion across the retina during spontaneous swimming. Perhaps supported by a largely hardwired optic tectum, this retinal motion may have been sufficient to drive the refinement of experience-dependent motion-sensing circuits in the retina^7,8,44^. In any case, the similar improvements for larvae exposed to static and moving stimuli—also reflected in our measurements of PSD-95 density—rule out the possibility that development of the spatial-frequency tuning function as indexed by the OMR reflected motor improvements from repeated invocation of the response.

We also found an experience-dependent increase in PSD-95 density in the IPL, which may be a subcellular marker of visual development. With visual experience, activity of neurons in the CNS induces up-regulation of BDNF synthesis^52^. Lack of BDNF expression in the developing rat retina can impair visual acuity^53^. This molecule plays a vital role in long-term potentiation (LTP) of neural responses and synaptic consolidation, which require high-frequency stimulation of excitatory inputs from the activation of NMDA receptors^54^. This activation also induces LTP at excitatory synapses for the experience-dependent refinement of developing retinal circuits in larval zebrafish^55^. Importantly, NMDA receptors rely on PSD-95 to anchor them on the post-synaptic membrane^15^, which may link PSD-95 to the BDNF-dependent LTP induced by early visual experience. Over-expression of PSD-95 can stabilise young synaptic contacts and promote excitatory synaptic maturation^16,17,56^. These results together suggest that PSD-95 plays a role in experience-dependent visual development. Future studies will clarify the molecular pathway that gives rise to increased PSD-95 density with visual experience. We also observed that variability in IPL PSD-95 density decreased for naïve larvae from 5 to 7 dpf, which may reflect increasing stability of PSD-95 expression with age even in the absence of visual experience. Quantifying PSD-95 density in the IPL provides a gross measure of overall synaptic changes; further investigations may reveal whether more subtle experience-dependent changes in synaptic density or distribution occur in certain neural subtypes, including direction-selective ganglion cells and starburst amacrine cells^57,58^, which are particularly important for processing information for motion detection.

The present study exemplifies some of the advantages of the larval zebrafish OMR in studying visual function and development. OMR measurement allows analysis of visual behaviour in relatively large samples compared to other assays such as the optokinetic response (OKR). For example, in our setup, every measurement in an OMR trial is averaged from ~50 larvae; this is advantageous in the context of rapid development, which can introduce considerable variability among larvae of the same age. Quantifying visual sensitivity at a given spatial frequency through swimming distance in response to a high-contrast stimulus—rather than calculating thresholds by varying contrast—improves efficiency by significantly reducing the number of experimental sessions required to measure the sensitivity of spatial vision. Furthermore, the OMR can be used to measure higher-order visual processing in zebrafish, targeting functions mediated by whole-brain visual circuits^59,60^. Using OMR in larval zebrafish, researchers can interrogate diverse visual-processing networks throughout the whole brain, especially when coupled with techniques such as two-photon calcium imaging^60^.

## Conclusions

The present study provides robust evidence of experience-dependent development of visual sensitivity in larval zebrafish from 5 to 7 dpf. This development is primarily driven by exposure to light and form, even in the absence of the explicit stimulus motion (i.e., non-self-generated motion) required for development of neural direction selectivity in other species^2,51^. PSD-95 density in the IPL was increased in 7-dpf larvae with visual experience; we found no evidence of a similar increase from 5 to 7 dpf in visually naïve larvae. These histological results support our OMR findings and suggest that visual exposure can up-regulate PSD-95 expression for refining synaptic connections, and may thus improve visual behaviour. Our results also contribute to a growing body of work demonstrating the utility of the larval OMR assay in experimental visual neuroscience.

## Supplementary information

Supplementary Information

## Data Availability

The datasets supporting this article are available on the Open Science Framework (https://osf.io/8emn7/).
