# Supplementary material for: Experience-dependent development of visual sensitivity in larval zebrafish
Source: Sci Rep. 2019 Dec 12;9:18931. doi: 10.1038/s41598-019-54958-6 (PMC6908733; doi:10.1038/s41598-019-54958-6)
Supplement: Supplementary file 1 — Supplementary Information [file 41598_2019_54958_MOESM1_ESM.pdf]

Supplementary information for
Experience-dependent development of visual sensitivity in larval zebrafish

Jiaheng Xie, Patricia R. Jusuf, Bang V. Bui, Patrick T. Goodbourn*

*Correspondence to: p.goodbourn@unimelb.edu.au

- Supplementary Figures S1–S3
- Supplementary Tables S1–S3

The datasets supporting this article are available on the Open Science Framework (https://osf.io/8emn7/).

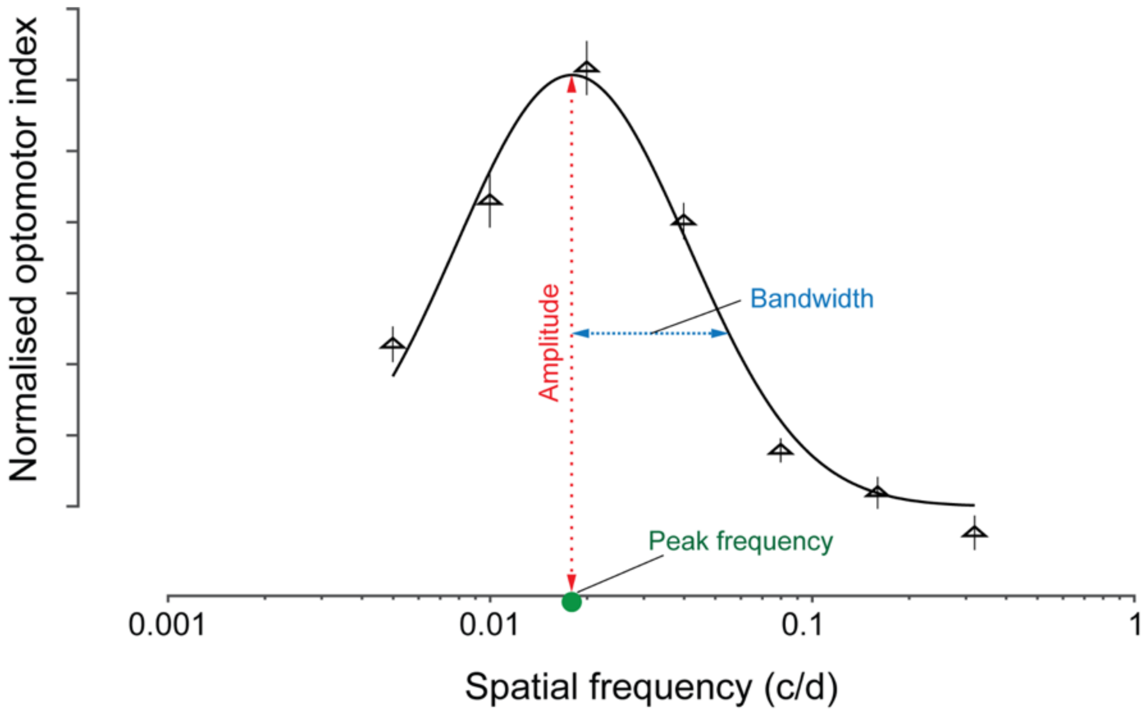

Supplementary Figure S1. Sample spatial-frequency tuning function from larval zebrafish optomotor response (OMR). The black curve represents a three-parameter log-Gaussian function, which provides an excellent fit to the data. The three free parameters of the fit are depicted: amplitude (i.e., height of the peak) is shown as a red dashed line; bandwidth (i.e., standard deviation) is shown as a blue dashed line; and peak frequency (i.e., the spatial frequency at peak amplitude) is shown as a green point on the x axis.

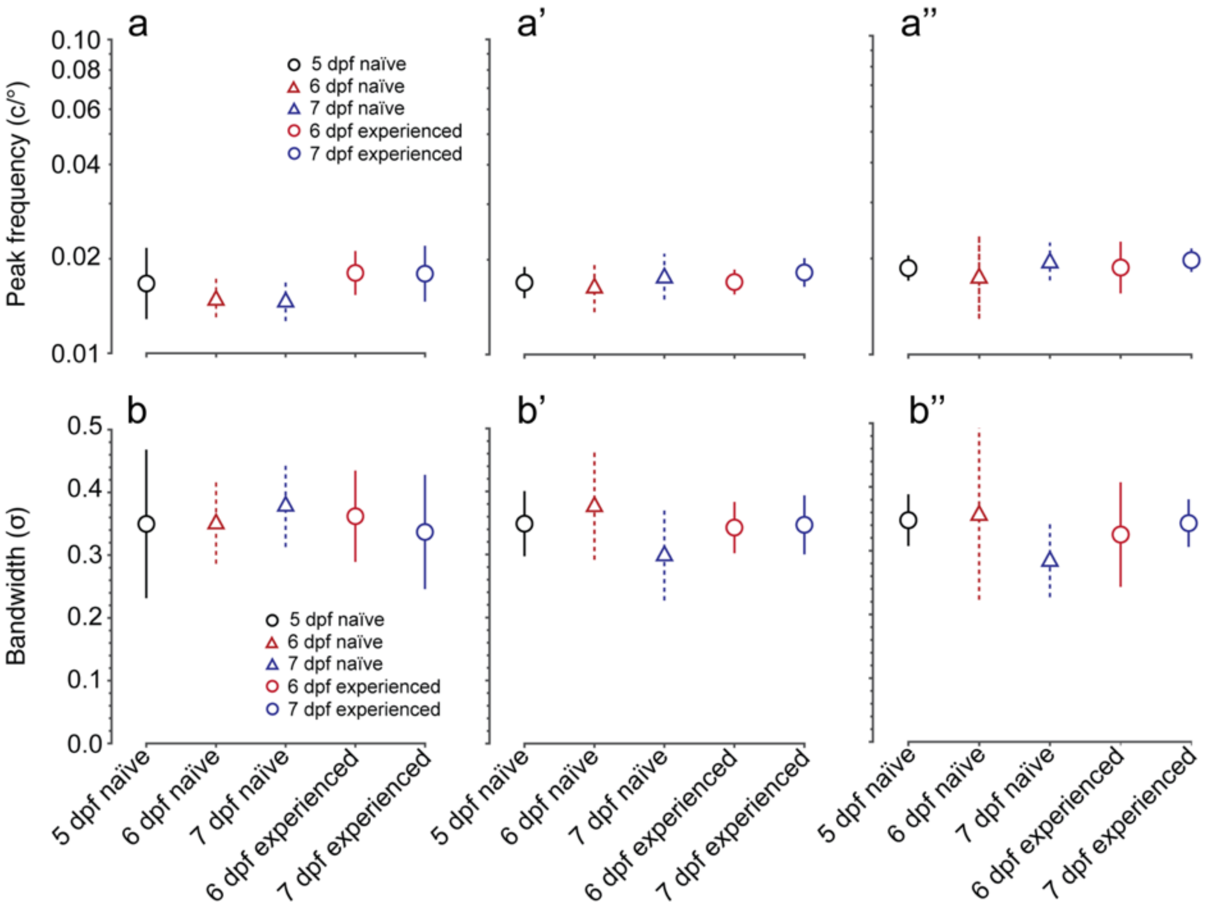

Supplementary Figure S2. Peak frequencies and bandwidths of spatial-frequency tuning functions for visually naïve and experienced larvae from 5 to 7 dpf. The upper row shows peak frequencies for stimulus speeds of (a) 25 °/s, (a') 50 °/s and (a'') 100 °/s. The lower row shows bandwidths at (b) 25 °/s, (b') 50 °/s and (b'') 100 °/s. Black circles, red triangles and blue triangles represent 5, 6, and 7 dpf visually naïve larvae, respectively. Red and blue circles represent 6- and 7-dpf visually experienced larvae, respectively. Error bars show the 95% confidence intervals around the fitted parameter. No differences between groups were significant ($P < .05$) after Bonferroni correction.

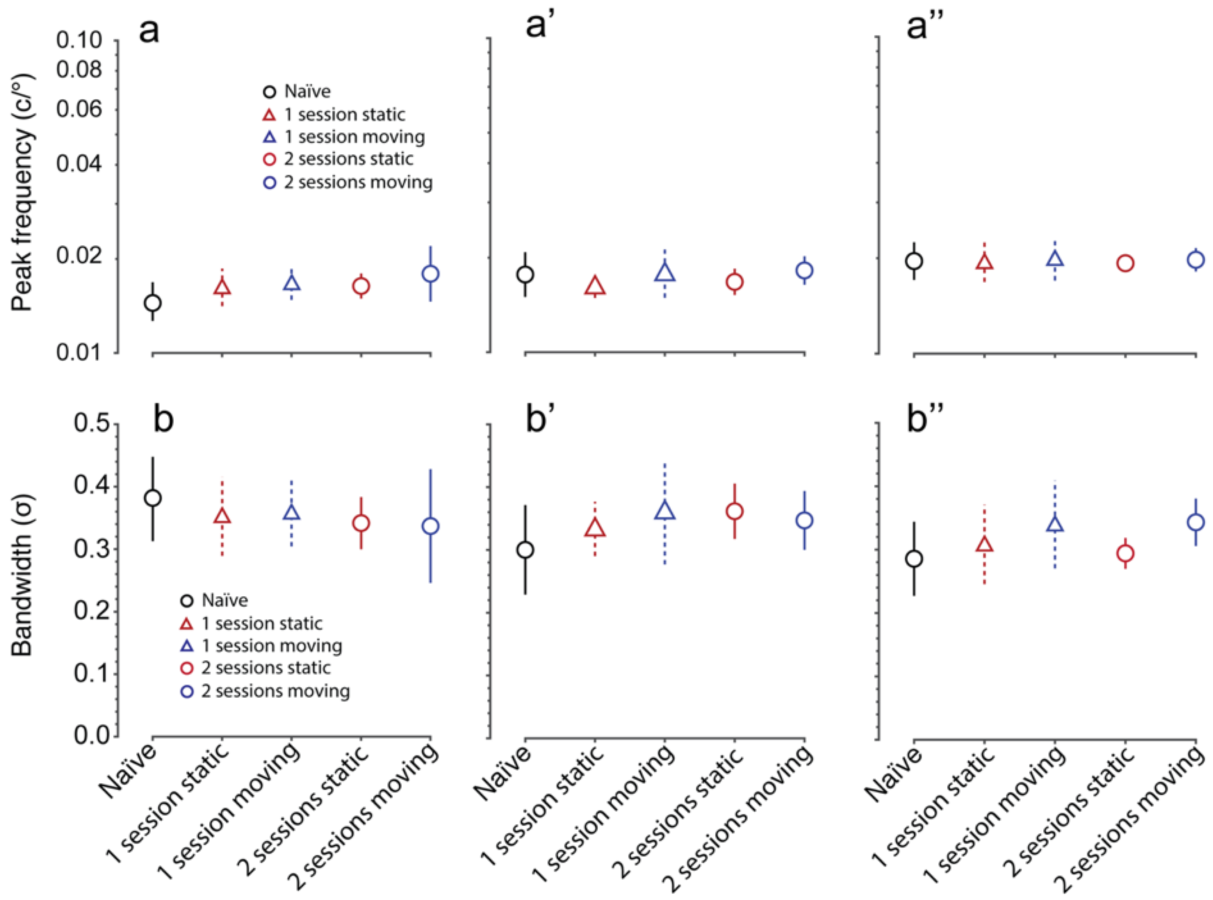

Supplementary Figure S3. Peak frequencies and bandwidths of spatial-frequency tuning functions for visually naïve and experienced larvae at 7 dpf. The upper row shows peak frequencies for stimulus speeds of (a) 25 °/s, (a') 50 °/s and (a'') 100 °/s. The lower row shows bandwidths at (b) 25 °/s, (b') 50 °/s and (b'') 100 °/s. Black circles, red triangles, blue triangles, red circles and blue circles indicate naïve, 1-session static, 1-session moving, 2-sessions static and 2-sessions moving groups, respectively. Error bars show the 95% confidence intervals around the fitted parameter. No differences between groups were significant ($P < .05$) after Bonferroni correction.

Supplementary Table S1. Statistical comparison of spatial-frequency tuning functions for visually naïve and experienced larvae from 5 to 7 dpf.

	25 °/s	50 °/s	100 °/s
Omnibus comparison (all parameters)	$F(12,20) = 2.34$<br>$^{*}P = .045$	$F(12,20) = 3.08$<br>$^{*}P = .013$	$F(12,20) = 3.95$<br>$^{**}P = .003$
Omnibus amplitude comparison	$F(4,20) = 3.59$<br>$^{*}P = .023$	$F(4,20) = 4.95$<br>$^{**}P = .006$	$F(4,20) = 6.44$<br>$^{**}P = .002$
Omnibus peak frequency comparison	$F(4,20) = 1.97$<br>$P = .137$	$F(4,20) = 0.70$<br>$P = .604$	$F(4,20) = 0.50$<br>$P = .738$
Omnibus bandwidth comparison	$F(4,20) = 0.26$<br>$P = .898$	$F(4,20) = 1.39$<br>$P = .272$	$F(4,20) = 0.78$<br>$P = .551$

5-, 6- and 7-dpf visually naïve groups performed 42, 30 and 30 trials per data point, respectively; 6- and 7-dpf visually experienced groups each performed 42 trials per data point.
*$P < .05$ after Bonferroni correction; **$P < .01$ after Bonferroni correction.

Supplementary Table S2. Statistical comparison of spatial-frequency tuning functions for visually naïve and experienced larvae at 7 dpf.

	25 °/s	50 °/s	100 °/s
Omnibus comparison (all parameters)	$F(12,20) = 1.40$<br>$P = .244$	$F(12,20) = 2.30$<br>$^{*}P = .048$	$F(12,20) = 5.90$<br>$^{***}P < .001$
Omnibus amplitude comparison	$F(4,20) = 2.47$<br>$P = .078$	$F(4,20) = 1.72$<br>$P = .186$	$F(4,20) = 8.53$<br>$^{***}P < .001$
Omnibus peak frequency comparison	$F(4,20) = 1.62$<br>$P = .209$	$F(4,20) = 0.83$<br>$P = .523$	$F(4,20) = 0.11$<br>$P = .979$
Omnibus bandwidth comparison	$F(4,20) = 0.42$<br>$P = .793$	$F(4,20) = 1.15$<br>$P = .361$	$F(4,20) = 1.90$<br>$P = .149$

Naïve, 1-session static, 1-session moving, 2-sessions static and 2-sessions moving groups performed 30, 24, 30, 30 and 42 trials per data point, respectively.
$^{*}P < .05$ after Bonferroni correction; $^{***}P < .001$ after Bonferroni correction.

Supplementary Table S3. Statistical comparisons of post-synaptic density 95 (PSD-95) in the retina inner plexiform layer of visually naïve and experienced larvae from 5 to 7 dpf.

Comparison between groups			$BF_{10,\ Uncorrected}$
5 dpf naïve	vs	6 dpf naïve	0.39
		7 dpf naïve	0.39
		7 dpf 2-sessions static	1.94
		7 dpf 2-sessions moving	1.66
6 dpf naïve	vs	7 dpf naïve	0.41
		7 dpf 2-sessions static	13.2**
		7 dpf 2-sessions moving	5.09*
7 dpf naïve	vs	7 dpf 2-sessions static	7.22*
		7 dpf 2-sessions moving	3.45*
7 dpf 2-sessions static	vs	7 dpf 2-sessions moving	0.37

12 retinae were analysed from each group, except for 6-dpf visually naïve larvae (10 retinae)
*Bayes Factor (BF) > 3; **BF > 10.
